# Interferon-Induced Transmembrane Protein 1 Restricts Replication of Viruses That Enter Cells via the Plasma Membrane

**DOI:** 10.1128/JVI.02003-18

**Published:** 2019-03-05

**Authors:** S. E. Smith, D. C. Busse, S. Binter, S. Weston, C. Diaz Soria, B. M. Laksono, S. Clare, S. Van Nieuwkoop, B. G. Van den Hoogen, M. Clement, M. Marsden, I. R. Humphreys, M. Marsh, R. L. de Swart, R. S. Wash, J. S. Tregoning, P. Kellam

**Affiliations:** a Wellcome Trust Sanger Institute, Wellcome Trust Genome Campus, Hinxton, United Kingdom; b Mucosal Infection and Immunity Group, Section of Virology, Imperial College London, St. Mary’s Campus, London, United Kingdom; c Department of Viroscience, Erasmus MC, Rotterdam, The Netherlands; d MRC Laboratory for Molecular Cell Biology, University College London, London, United Kingdom; e Division of Infection and Immunity/Systems Immunity University Research Institute, Cardiff University, Cardiff, United Kingdom; f Kymab Ltd., Babraham Research Campus, Cambridge, United Kingdom; University of Pittsburgh School of Medicine

**Keywords:** IFITM1, innate immunity, paramyxovirus, pneumovirus, restriction factor

## Abstract

The acute antiviral response is mediated by a family of interferon-stimulated genes (ISGs), providing cell-intrinsic immunity. Mutations in genes encoding these proteins are often associated with increased susceptibility to viral infections. One family of ISGs with antiviral function is the interferon-inducible transmembrane proteins (IFITMs), of which IFITM3 has been studied extensively. In contrast, IFITM1 has not been studied in detail. Since IFITM1 can localize to the plasma membrane, we investigated its function with a range of enveloped viruses thought to infect cells by fusion with the plasma membrane. Overexpression of IFITM1 prevented infection by a number of *Paramyxoviridae* and *Pneumoviridae*, including respiratory syncytial virus (RSV), mumps virus, and human metapneumovirus (HMPV). IFITM1 also restricted infection with an enveloped DNA virus that can enter via the plasma membrane, herpes simplex virus 1 (HSV-1). To test the importance of plasma membrane localization for IFITM1 function, we identified blocks of amino acids in the conserved intracellular loop (CIL) domain that altered the subcellular localization of the protein and reduced antiviral activity. By screening reported data sets, 12 rare nonsynonymous single nucleotide polymorphisms (SNPs) were identified in human *IFITM1*, some of which are in the CIL domain. Using an *Ifitm1^−/−^* mouse, we show that RSV infection was more severe, thereby extending the range of viruses restricted *in vivo* by IFITM proteins and suggesting overall that IFITM1 is broadly antiviral and that this antiviral function is associated with cell surface localization.

**IMPORTANCE** Host susceptibility to viral infection is multifactorial, but early control of viruses not previously encountered is predominantly mediated by the interferon-stimulated gene (ISG) family. There are upwards of 300 of these genes, the majority of which do not have a clearly defined function or mechanism of action. The cellular location of these proteins may have an important effect on their function. One ISG located at the plasma membrane is interferon-inducible transmembrane protein 1 (IFITM1). Here we demonstrate that IFITM1 can inhibit infection with a range of viruses that enter via the plasma membrane. Mutant IFITM1 proteins that were unable to localize to the plasma membrane did not restrict viral infection. We also observed for the first time that IFITM1 plays a role *in vivo*, and *Ifitm1^−/−^* mice were more susceptible to viral lung infection. These data contribute to our understanding of how ISGs prevent viral infections.

## Abstract (precis)

Host susceptibility to viral infection is multifactorial, but early control of viruses not previously encountered is predominantly mediated by the interferon-stimulated gene (ISG) family. There are upwards of 300 of these genes, the majority of which do not have a clearly defined function or mechanism of action. The cellular location of these proteins may have an important effect on their function. One ISG located at the plasma membrane is interferon-inducible transmembrane protein 1 (IFITM1). Here we demonstrate that IFITM1 can inhibit infection with a range of viruses that enter via the plasma membrane. Mutant IFITM1 proteins that were unable to localize to the plasma membrane did not restrict viral infection. We also observed for the first time that IFITM1 plays a role *in vivo*, and *Ifitm1^−/−^* mice were more susceptible to viral lung infection. These data contribute to our understanding of how ISGs prevent viral infections.

## INTRODUCTION

Intrinsic immunity is the ability of infected and bystander cells to restrict infection prior to the recruitment of innate or adaptive immune cells (1). This intrinsic immune response is in part mediated by proteins encoded by interferon (IFN)-stimulated genes (ISGs). There are over 300 of these genes that are upregulated in response to type I, II, and III interferons (2). Although the functions and modes of action of a few of these genes have been studied in detail, many remain to be functionally characterized (3). Nevertheless, the importance of ISGs in defense against various pathogens is demonstrated by increased disease severity associated with single nucleotide polymorphisms (SNPs) in genes encoding ISGs, including *IFITM3* (4), *MDA5* (5), *OAS-1* (6), and *Mx1* (6–8).

One family of ISGs that functions as broad-spectrum inhibitors of viral replication is the interferon-inducible transmembrane protein (IFITM) family. IFITMs are functionally conserved across many species, including birds (9–12), pigs (13, 14), and bats (13). In most cases, this family of restriction factors blocks infection during virus entry into cells (15), although additional mechanisms have been proposed (16, 17). It is proposed that these very similar proteins arose by gene duplication events (18), but their maintenance across many species suggests that they have distinct functions or specializations. While IFITM2 and IFITM3 share 90% of their amino acids, IFITM1 shares only 74% with IFITM3, due largely to an N-terminal deletion of 21 amino acids. Research into IFITM proteins has mainly focused on IFITM3 and investigation of its ability to inhibit entry and replication of RNA viruses, including influenza virus (19–22), dengue virus (20, 23), Zika virus (24), respiratory syncytial virus (RSV) (25), Semliki Forest and Sindbis viruses (26), and murine cytomegalovirus (mCMV) (27). Fewer studies have been performed on IFITM1, which can restrict infection by a number of RNA viruses, including hepatitis C virus (28, 29), sheep Jaagsiekte virus (30), HIV (31), Zika virus (24), and influenza viruses (20) but not Rift Valley fever virus (32), Sindbis virus, or Semliki Forest virus (26). IFITM1 has no reported antiviral activity against the nonenveloped DNA viruses human papillomavirus and adenovirus (33).

Interestingly, the IFITM proteins differ in their subcellular localizations when expressed individually: IFITM1 is found primarily on the cell surface (10, 34), IFITM2 is found in late endosomes, and IFITM3 is found mainly in early endosomes (34). The function of IFITM1 may thus be linked to its abundance in the plasma membrane. Indeed, mutations that increase IFITM1 cell surface expression lead to increased restriction of HIV-1_NL4-3_ infection compared to wild-type (WT) IFITM1 (31). Moreover, mutations in IFITM1 that prevent its binding to the vesicular transport adaptor protein AP3 reduced inhibition of viral replication (35).

Greater examination of the range of viruses restricted by IFITM1 and the effect of engineered and naturally occurring mutations in IFITM1 is required to further understand the mechanism of IFITM1 viral restriction. Here we show that *in vitro*, IFITM1 inhibits infection by several RNA viruses that enter via the plasma membrane, including mumps virus, RSV, human metapneumovirus (HMPV), and a DNA virus, herpes simplex virus 1 (HSV-1). Furthermore, we show that otherwise healthy *Ifitm1^−/−^* mice experience more-severe RSV infection than wild-type mice. However, mCMV infection, which is altered in *Ifitm3^−/−^* mice, was no more severe in *Ifitm1^−/−^* mice. This suggests that IFITM1 has antiviral activity that is distinct from that of IFITM3.

## RESULTS

### Restriction of *Paramyxoviridae* and *Pneumoviridae* by human IFITM1.

**FIG 1:**
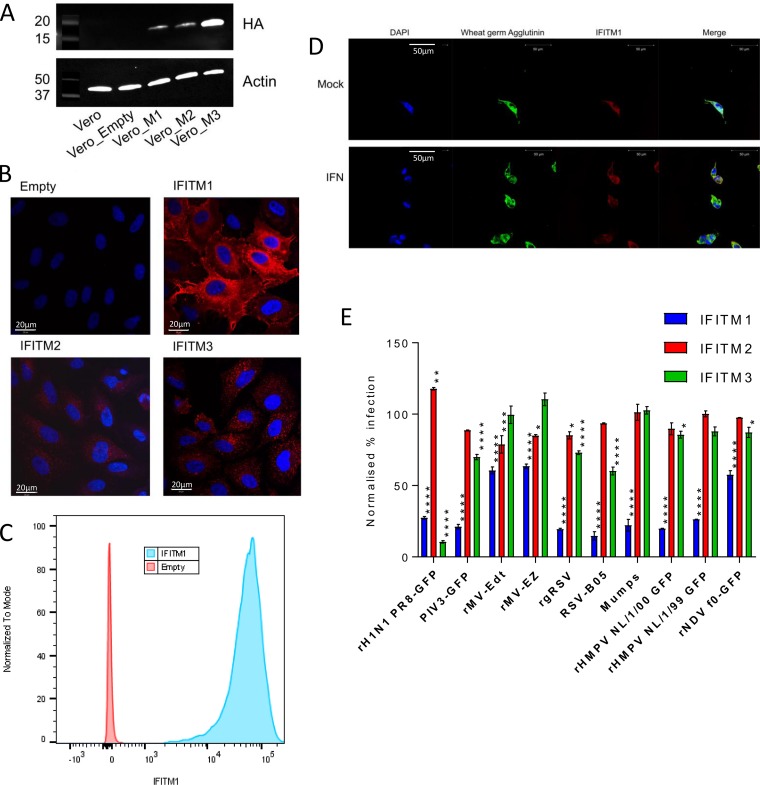
IFITM1 restricts replication of a wide range of RNA viruses *in vitro*. (A) Overexpression of IFITM proteins in Vero cells detected by Western blotting using an antibody to the C-terminal HA tag, including IFITM1 (Vero_M1), IFITM2 (Vero_M2), and IFITM3 (Vero_M3). Detection of β-actin expression was used as a control, and Vero Empty is the non-IFITM expression vector negative control. (B) Localization of different IFITM proteins was detected by confocal microscopy using an antibody to an inserted HA tag (red). Nuclei are stained with DAPI (blue). (C) Analysis of surface expression of HA-tagged IFITM1 by flow cytometry on nonfixed and nonpermeabilized cells. (D) Colocalization of IFITM1 (red) and wheat germ agglutinin (green) was detected by confocal microscopy. Nuclei are stained with DAPI (blue). (E) Transduced Vero cells were seeded in 24-well plates and infected at a range of MOIs with influenza A virus A/Puerto Rico/8/1934 (H1N1 PR8), parainfluenza virus 3 (PIV3), measles virus (rMV-Edt and rMV-EZ), respiratory syncytial virus (rgRSV and RSV-B05), mumps virus (mumps), human metapneumovirus NL/1/00-GFP (rHMPV NL1/1/00), human metapneumovirus NL/1/99-GFP (rHMPV NL/1/99), and Newcastle disease virus (rNDV). At 24 hpi, cells were fixed, and the level of infection of each cell line was measured by flow cytometry. *, *P* < 0.05; **, *P* < 0.01; ***, *P* < 0.001; ****, *P* < 0.0001 (by analysis of variance [ANOVA], compared to cells transduced with an empty vector control [*n* = 3]).

Previous studies have demonstrated that IFITM1 can restrict infection by some RNA viruses (20, 24, 28, 29, 31). Given our previous findings that IFITM1 is preferentially localized to the cell surface (34), we sought to extend these findings to the *Paramyxoviridae* and *Pneumoviridae*, which are negative-stranded RNA viruses that are thought to enter cells at the plasma membrane. These families include viruses of clinical importance, such as measles virus (MV), mumps virus, and RSV. Lentiviral vectors were used to stably overexpress IFITM1, -2, or -3 proteins in Vero cells, which are permissive to infection with the above-described viruses. The proteins were HA tagged to enable detection, and transfection led to detectable expression in the cells (Fig. 1A). IFITM1 expression in Vero cells was observed throughout the cell, with a concentration in the perinuclear space and, unlike IFITM2 and IFITM3, which were localized internally and formed a punctate pattern, distinct expression at the cell surface (Fig. 1B). Cell surface expression of IFITM1 was confirmed by flow cytometry analysis on nonfixed and nonpermeabilized cells (Fig. 1C) and colocalization with wheat germ agglutinin (Fig. 1D). This pattern of expression was consistent with previous studies which further confirmed cell surface expression of IFITM1 in these cell lines using additional assays (34).

IFITM1- to -3-transduced Vero cells were infected with different members of the *Paramyxoviridae* and *Pneumoviridae*, and infection was compared to that in Vero cells transduced with an empty vector. IFITM1 restricted infection of all the viruses tested, including parainfluenza virus (PIV), RSV, HMPV, Newcastle disease virus (NDV), and mumps virus (Fig. 1E). There was a small, but significant, effect on measles virus and NDV replication. IFITM2 had no impact on any of the viruses tested. As shown previously, only RSV was restricted by IFITM3 (25, 36). Comparisons were made to influenza virus (an orthomyxovirus), which confirmed that both IFITM1 and IFITM3 are able to restrict influenza A virus infection effectively.

### IFITM1 restricts HSV-1 infection.

**FIG 2:**
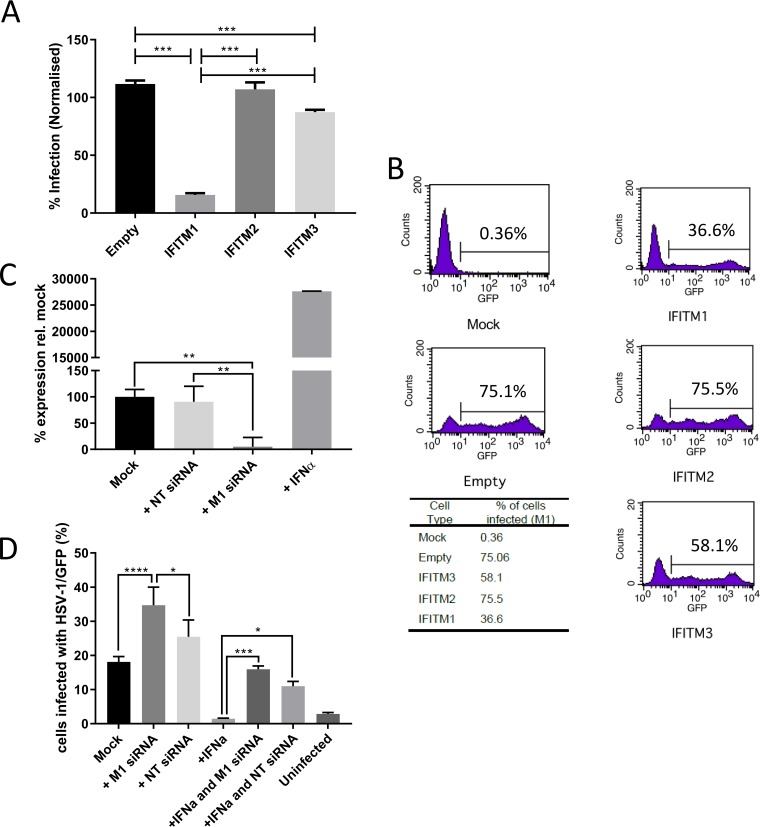
IFITM1 restricts HSV-1 infection. (A) A549 cell lines stably expressing an empty vector, IFITM3, IFITM2, or IFITM1 were generated using lentiviruses. The cell lines were infected with HSV-1/GFP (MOI of 5; *n* = 3). GFP expression was measured on a Cellomics ArrayScan instrument at 7 hpi and normalized to infection levels in untransduced A549 cells. (B) Transduced A549 cells were infected with HSV-1/GFP at an MOI of 0.01. Cells were harvested at 44 hpi, and GFP expression was detected by flow cytometry. (C) MRC-5 cells were treated with IFN-α2a, siRNA targeting IFITM1, or nontargeting siRNA or mock treated. Total RNA was extracted, and the expression of IFITM1 was measured. Data are presented as percentages of expression relative to the mock-treated cells ± standard deviations (SD) (*n* = 3). (D) Treated MRC-5 cells were infected with HSV-1/GFP at an MOI of 0.5 for 7 h, and GFP expression was measured on a Cellomics ArrayScan instrument (means ± SD). Significance was determined by ANOVA. *, *P* < 0.05; **, *P* < 0.01; ***, *P* < 0.001; ****, *P* < 0.0001.

These data support an antiviral role for IFITM1 against a selected group of RNA viruses. Previous studies suggested that IFITM1 has no significant impact on DNA viruses such as papillomaviruses and adenoviruses. However, both of these are nonenveloped viruses, which, for the most part, are not restricted by IFITM proteins. To explore the role of IFITM1 in enveloped DNA viruses that can enter the cell via the plasma membrane, we looked at the effect of IFITM1 expression on infection by HSV-1, a member of the *Alphaherpesvirinae*. A549 human fibroblasts were transduced with lentiviruses coding for human IFITM1, IFITM2, or IFITM3 proteins. Transfected cells were infected with HSV-1 expressing green fluorescent protein (HSV-1/GFP) at a multiplicity of infection (MOI) of 5. Quantitative fluorescence microscopy showed that at 7 h postinfection (hpi), 16% of cells expressing IFITM1 were infected by HSV-1, compared to 87% and 107% of cells expressing IFITM3 and IFITM2, respectively (values normalized to those for untransduced cells) (Fig. 2A). These findings were supported by flow cytometry analysis of a multicycle HSV-1 infection. After 44 h, the level of HSV infection (MOI of 0.01) in IFITM1-expressing A549 cells was 36.6%, compared to 75.5% and 58.1% for IFITM2 and IFITM3, respectively, and 75.1% infection of control empty vector-transduced cells (Fig. 2B).

To confirm the role of IFITM1, we looked at the effect of gene knockdown. A SMARTpool of small interfering RNAs (siRNAs) targeting human IFITM1 reduced the expression of IFITM1 mRNA in MRC-5 cells by 96% (−4.72-log_2_ reduction) compared to interferon alpha treatment (Fig. 2C). The nontargeting control had some effect on IFITM1 transcription. Pretreatment with the human IFITM1-specific siRNAs increased HSV-1 infection, compared to untreated cells and a nontargeting siRNA control (Fig. 2D). Pretreatment with IFN-α2a substantially reduced HSV-1 infection, but the addition of siRNA against IFITM1 to IFN-α2-treated cells negated the effect of IFN-α2a. Collectively, these data suggest that IFITM1 is an important part of the IFN response to HSV-1 infection.

### Amino acids in the CIL domain of IFITM1 are important for restriction.

**FIG 3:**
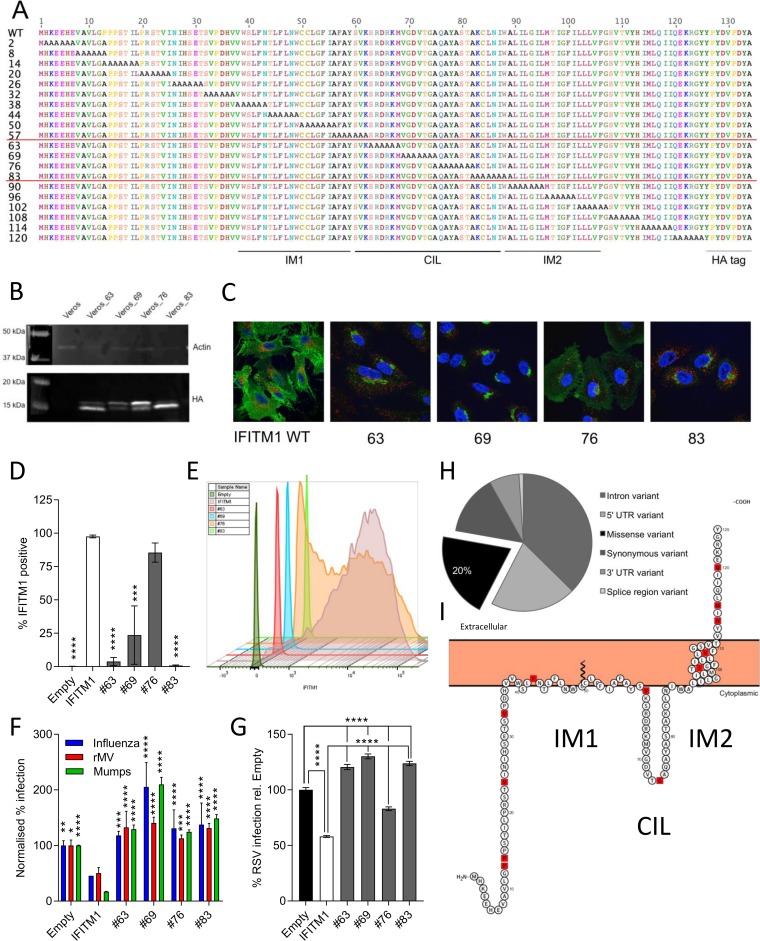
IFITM1 domains necessary for membrane localization and virus restriction. (A) Twenty mutant human IFITM1 proteins were designed by mutating sequential blocks of 6 amino acids to alanine from the N to the C terminus of the protein. (B) A selection of these proteins with alanine blocks in the CIL domain were overexpressed in Vero cells using lentiviral constructs and puromycin selection. Expression of the HA-tagged protein was detected by Western blotting. (C) Localization of mutant protein expression was compared to that of wild-type human IFITM1. HA-tagged proteins are shown in green (anti-HA-488), and LAMP1 expression is shown in red. (D) Analysis of surface expression of HA-tagged CIL mutants of IFITM1 by flow cytometry on nonfixed and nonpermeabilized cells. (E) Representative plot showing relative surface expression of CIL mutants. (F) Vero cells were also infected with influenza virus, measles virus (rMV), or mumps virus at an MOI of 1, and the level of infection of each cell line was measured by fluorescence microscopy at 24 hpi (Cellomics ArrayScan). (G) Mutant IFITM1 proteins were also overexpressed in A549 cells. Cells were infected with rgRSV (MOI of 0.8) for 24 h prior to analysis of infectivity by flow cytometry (*n* = 3). (H) A total of 93 single nucleotide polymorphisms (SNPs) in the IFITM1 gene were identified. UTR, untranslated region. (I) Location of these SNPs in the human IFITM1 protein marked in red. *, *P* < 0.05; **, *P* < 0.01; ***, *P* < 0.001; ****, *P* < 0.0001 (by ANOVA, with significance relative to wild-type IFITM1 [*n* = 3]).

The current model of the IFITM1 structure establishes it as having its short N-terminal domain in the cytoplasm, two membrane domains linked by a conserved intracellular loop (CIL) exposed to the cytoplasm, and the C-terminal domain exposed on the cell surface (34). In order to determine the amino acids that are important for IFITM1 localization and virus restriction, we generated a panel of 20 cell lines expressing mutant proteins with consecutive substitutions of six alanines, starting from the second N-terminal amino acid (Fig. 3A). These mutated proteins were expressed in A549 cells (Fig. 3B), and their localizations were established by immunofluorescence using an antibody against the C-terminal HA tag (Fig. 3C). Since the CIL domain is predicted to be exposed to the cytoplasm (34), mutations in this domain were not expected to alter IFITM1 localization. However, in the 6-alanine mutants AA-63 (i.e., mutant with a 6-alanine substitution at amino acid 63 [Fig. 3A]), AA-69, and AA-83, IFITM1 was not seen at the cell surface but was primarily associated with LAMP1 (a marker for late endosomes and lysosomes)-negative intracellular membranes. Interestingly, AA-76 IFITM1 appeared to show both intracellular and surface localizations. The loss of cell surface expression with mutation of the CIL domain was confirmed by flow cytometry on nonfixed and nonpermeabilized cells; the wild-type protein was detected at significantly higher levels on the cell surface than the AA-63, -69, or -83 mutant (Fig. 3D and E), and there was no significant difference in surface expression of the AA-76 mutant and the wild type. However, there was a decrease in the median fluorescence intensity (MFI) of A549 AA-76 cells, suggesting that there are reduced levels of cell surface IFITM1 expression (Fig. 3E).

To determine whether mutations in the CIL domain affected function, cells expressing wild-type IFITM1, a negative-control empty vector, or IFITM1 with 6 alanines inserted at amino acid 63, 69, 76, or 83 were infected with influenza virus, measles virus, mumps virus, or RSV. As described above, overexpression of wild-type IFITM1 reduced infection for all tested viruses, relative to empty cells (Fig. 3F and G). Cell lines expressing the IFITM1 AA-63, -69, -76, or -83 mutant showed increased infection relative to cells expressing wild-type IFITM1, suggesting an impairment of IFITM-mediated restriction. Interestingly, IFITM1 AA-76, which was seen to maintain some cell surface expression, unlike the other mutant proteins, was still able to restrict RSV by ∼20% (Fig. 3G). However, this was still a significant reduction in restriction compared to that observed with overexpression of the wild-type protein. Together, the infectivity and immunofluorescence data indicate that the CIL domain influences IFITM1 localization and is important for IFITM1’s antiviral activity. This may suggest that IFITM1 function is dependent upon its localization to the cell surface, rather than intracellular membranes, which requires an intact CIL domain.

Having observed that IFITM1 can restrict infection by enveloped RNA and DNA viruses and that sequence alterations in the CIL domain effectively impair function, we investigated whether there are common SNPs in the IFITM1 gene. To map these SNPs, variants were identified in *IFITM1* from the 1000Genomes phase 3 data set (2,504 people), the UK10K control cohorts (2,453 people), and 11 UK10K disease cohorts (6,053 people). In total, 93 SNPs were identified across the entire gene (Fig. 3H). Of these, 12 (20%) resulted in nonsynonymous substitutions, but all SNPs were very rare and were rarely seen in multiple cohorts (Table 1). The exception is SNP rs9667990 (P13A), which is seen in the vast majority of individuals; it is therefore likely that a proline at amino acid 13 was a rare amino acid substitution in the reference sequence and that alanine is the correct, common amino acid. The location of these nonsynonymous SNPs is shown across the entire IFITM1 protein (Fig. 3I).

**TABLE 1:** SNPs in the IFITM1 gene resulting in amino acid substitutions^*a*^

SNP	Amino acid change	Nucleotide change	GMAF		
			UK10K disease cohorts	UK10K controls	1000Genomes
rs9667990	P13A	CCA/GCA	1	1	1
COSM46151	P14S	CCC/TCC	0.00023		
rs374294080	V24M	GTG/ATG			0.00020
rs371803538	V33M	TGT/ATG			0.00020
rs764916857	F42L	TTC/TTG		0.00026	
rs373112031	V61M	GTG/ATG	0.00050		
rs200528039	G74R	GGG/AGG	0.00055		
rs557063411	I98T	ATT/ACT			0.00020
rs201082701	V105I	GTA/ATA			0.00040
rs199539158	H113R	CAT/CGT	0.00046	0.00026	
rs191154799	M115I	ATG/ATA			0.00040
rs572703137	Q120R	CAG/CGG			0.00020

a A total of 93 single nucleotide polymorphisms (SNPs) in the IFITM1 gene were identified from 11 UK10K disease cohorts, UK10K controls, and 1000Genomes data sets using custom scripts. Twelve SNPs result in an amino acid substitutions, shown in the table along with the minor allele frequencies (MAFs). GMAF, global minor allele frequency.

### RSV disease is more severe in mice lacking IFITM1.

**FIG 4:**
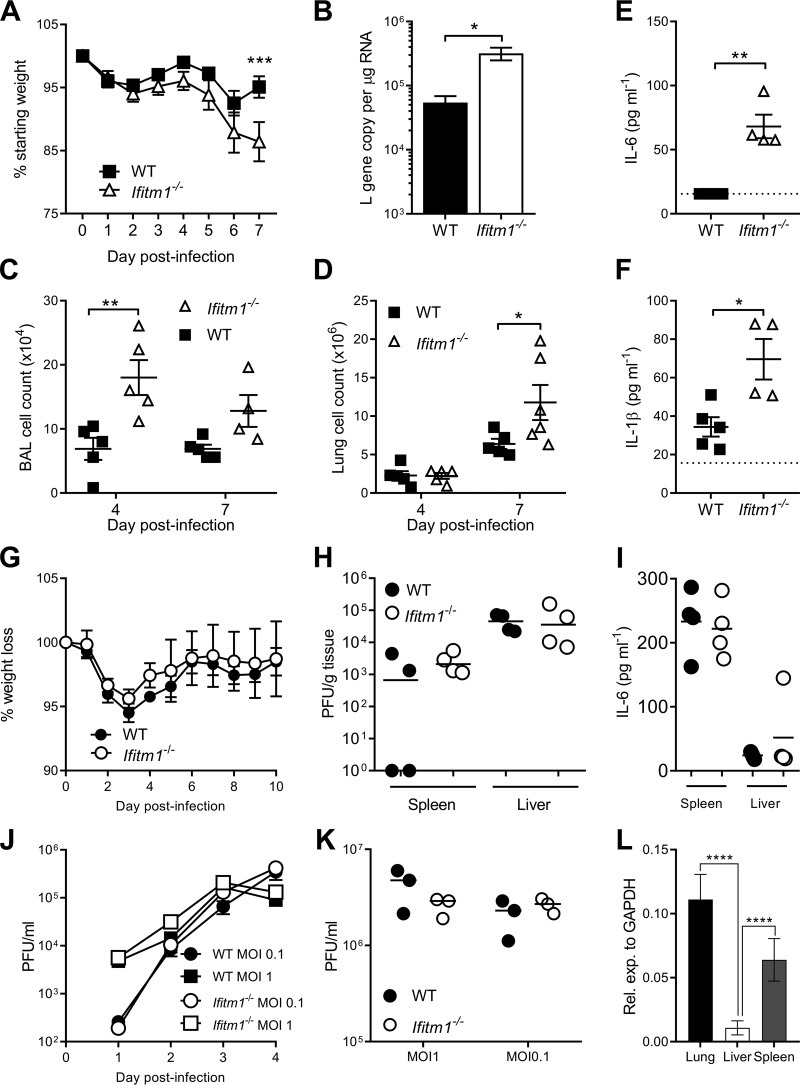
*Ifitm1^−/−^* mice increase RSV but not mCMV infection. Homozygous knockouts and wild-type mice were intranasally infected with 5 × 10^5^ PFU of RSV-A2. (A) Weight loss was measured over the course of 7 days. (B) RSV viral load was measured by quantitative RT-PCR for the RSV L gene at day 4 after infection. (C and D) Cells in airways (C) and lungs (D) after infection. BAL, bronchoalveolar lavage fluid. (E and F) Lungs were homogenized and centrifuged, and the supernatant was collected for IL-6 (E) and IL-1β (F) analyses 4 days after infection. Mean values are shown (*n* ≥ 5) (A and B). Points represent data for individual animals (C to E). (G and H) WT and *Ifitm1^−/−^* mice were infected with mCMV, weight loss was measured throughout (G), and after 4 days, the virus load in spleen and liver was measured by a plaque assay (H). (I) IL-6 concentrations in spleens and livers of mCMV-infected WT and *Ifitm1^−/−^* mice 4 days after infection. (J and K) BMDM cells (J) and MEFs (K) were infected with mCMV. (L) *Ifitm1* was quantified in lung, liver, and spleen of BALB/c mice (*n* = 5). GAPDH, glyceraldehyde-3-phosphate dehydrogenase. *, *P* < 0.05; **, *P* < 0.01; ***, *P* < 0.001; ****, *P* < 0.0001 (by ANOVA [A and L] or a *t* test [B to K]).

As IFITM1 affects viral replication *in vitro*, we wished to determine its role *in vivo. Ifitm1*^−/−^ mice and wild-type C57BL/6 mice were intranasally infected with RSV A2 and monitored daily for weight loss for 7 days after infection (Fig. 4A). *Ifitm1*^−/−^ mice showed significant weight loss on day 7 after infection compared to wild-type littermates (*P* < 0.05) (Fig. 4A). There was a significantly higher lung RSV viral load at day 4 after infection (Fig. 4B) and significantly more cells in the airways at day 4 (Fig. 4C) and in the lungs at day 7 (Fig. 4D) after infection. To determine the effect of IFITM1 on the inflammatory response, the lungs of all mice were homogenized, and the levels of interleukin-6 (IL-6) (Fig. 4E) and IL-1β (Fig. 4F) were compared between genotypes after RSV infection. The levels of both cytokines were significantly higher in infected knockout (KO) mice than in wild-type littermates.

To test the effect of IFITM1 in an established herpesvirus model, we infected WT and *Ifitm1*^−/−^ mice with the betaherpesvirus mCMV. IFITM1 deficiency did not impact virus-induced weight loss in this model (Fig. 4G). Moreover, in accordance with the reported lack of a role for IFITM1 in restriction of human cytomegalovirus (hCMV) replication (33, 37), mCMV replication was not increased in the absence of IFITM1 *in vivo* (Fig. 4H). Given that IFITM1 deficiency during RSV infection led to increased IL-6 production and *Ifitm3*^−/−^ mice suffer from IL-6-driven pathogenesis during mCMV infection (27), we assessed IL-6 production in mCMV-infected *Ifitm1*^−/−^ mice. In accordance with the unaltered weight loss observed in these mice, *Ifitm1* deficiency did not influence virus-induced cytokine production (Fig. 4I). As mCMV and RSV infect different tissues, we quantified basal *Ifitm1* expression in the lungs, liver, and spleens of wild-type BALB/c mice (Fig. 4L). Expression was detectable in each tissue but was significantly lower in the liver. However, expression levels were comparable between lung and spleen tissue. Furthermore, when bone marrow-derived myeloid (BMDM) cells and mouse embryonic fibroblasts (MEFs) from wild-type and KO mice were infected *ex vivo* with mCMV, there was no difference in viral titers (Fig. 4J and K).Thus, overall, these data demonstrate that IFITM1 does not influence the replication and associated pathogenesis of a betaherpesvirus *in vivo*.

## DISCUSSION

Here we demonstrate that IFITM1 has wide-ranging antiviral function, restricting the replication of RNA viruses from the *Paramyxoviridae* and *Pneumoviridae* families. Importantly, for the first time, we demonstrate the antiviral function of IFITM1 against a DNA virus, HSV-1. Our findings suggest that the cellular localization of the protein is critical for its function.

Sequential mutation of the CIL domain of IFITM1 revealed that this domain was essential in determining cellular localization and antiviral activity. Stably transduced Vero cells were found to express wild-type IFITM1 in a diffuse manner, likely on the cell surface but also potentially in the cytoplasm. Previous studies, including the initial study identifying IFITM1 (referred to as Leu-13 antigen and subsequently as CD225), have shown that IFITM1 is primarily expressed on the plasma membrane (38). Most subsequent studies have shown that IFITM1 has domains expressed on the cell surface using a range of assays, including cell surface biotinylation and antibody labeling in the absence of plasma membrane disruption (10, 28, 34, 39). However, some studies have suggested that IFITM1 is also expressed internally in vesicles distinct from either IFITM2 or -3, although they have not been specifically identified (32, 35). Here we find evidence for different cellular locations of IFITM1 with mutation of the CIL domain; interestingly, the AA-76 mutant showed the highest level of cell surface expression and the greatest reduction of RSV infection. A proposed mechanism of action for the IFITM proteins is that they alter the fluidity of cellular membranes, preventing fusion with the infecting virus envelope (21, 30). We suggest that IFITM1, unlike IFITM2 and IFITM3, primarily functions through alteration of the plasma membrane and, as such, is able to restrict viruses at this initial point in infection.

This is the first reported study describing viral infection in *Ifitm1*^−/−^ animals. Uninfected *Ifitm1*^−/−^ mice were phenotypically normal, as assessed by the Wellcome Trust Sanger Institute murine phenotyping pipelines. We observed a different phenotype depending on the virus infection: *Ifitm1*^−/−^ mice were more susceptible to RSV infection, as judged by viral RNA, cell infiltration, cytokine production, and body weight loss. However, no effect was seen in mCMV-infected animals. This may in part be due to differences in where the infection is localized, as there are lower levels of *Ifitm1* in the liver than in the lung. However, mCMV also establishes infection in the spleen after systemic administration (40), where there is comparable *Ifitm1* expression to the lung. It was previously shown that another member of the IFITM family, IFITM3, restricts RSV *in vivo* (25). Interestingly, IFITM3 has also been shown to restrict mCMV pathogenesis *in vivo*, and this is due to modulation of proinflammatory cytokine production rather than direct control of virus replication (27). The observation that IFITM1 did not influence mCMV pathogenesis in our experiments highlights fundamental functional differences between IFITM1 and IFITM3. Further studies of the immunoregulatory functions of IFITM3 and, possibly, IFITM1 will be informative.

Not all enveloped viruses are restricted by IFITM1. The differences in virus inhibition may reflect differences in the route by which the virus infects the cell. Some viruses may bypass IFITM1 at the plasma membrane; for example, mCMV enters certain cell types (e.g., myeloid cells) by endocytosis-dependent mechanisms (27). Furthermore, we cannot preclude the possibility that IFITM1 may restrict initial mCMV cell entry into other cells (e.g., fibroblasts) but that a subsequent, previously described proviral role for IFITM1 (37) may mask this effect in our assays.

Further investigation into how IFITM1 affects human susceptibility to viral infection is required. We have previously reported that SNPs in IFITM3 were associated with more-severe influenza virus infection (4). In the present study, we report a list of SNPs found in the *IFITM1* gene. In the 11,000 individuals screened, we identified 93 SNPs, 20% of which were rare nonsynonymous SNPs. Future studies will need to focus on how the protein interacts with viruses to prevent their entry into the cell. An improved understanding of the function of this ISG in the control of viral lung infection could also inform the design of novel antiviral strategies.

## MATERIALS AND METHODS

### Cell culture.

A549 cells (ATCC CCL-185) were grown in F-12 medium (Life Technologies), MRC-5 cells (ATCC CCL-171) were grown in Eagle's minimum essential medium (EMEM) (ATCC), and U2OS cells (ATCC HTB-96) were grown in McCoy’s medium (Life Technologies). Vero cells (catalog number 84113001; Sigma), HEp2 cells (ATCC CCL-23), and HEK293-T/17 cells (ATCC CRL-11268) were grown in Dulbecco’s modified Eagle’s medium (DMEM) (Life Technologies). All media were supplemented with 10% (vol/vol) fetal bovine serum (FBS) (Biosera).

### Overexpression studies.

Human IFITM1 wild-type and alanine-scanned gene sequences were synthesized (GeneArt; Life Technologies) for expression in human cells. Single amino acid changes were introduced using site-directed mutagenesis (QuikChange II XL; Agilent). All IFITM genes were cloned into the BamHI and NotI sites of the lentivirus vector pSIN-BN_puro (41), and sequences were confirmed by capillary sequencing (GATC Biotech). The wild-type human genes cloned were IFITM1, IFITM2, and IFITM3. The gene cassette was cloned into pSIN-BN along with a C-terminal HA tag to facilitate analysis of the expressed protein. Lentivirus vector stocks were made by three-plasmid transfection of HEK293-T/17 cells, grown to confluence in a 10-cm^2^ dish (10). The lentiviruses were used to transduce A549 or Vero cells and produced a mixed population of IFITM-expressing cells. Transduced cells were selected using puromycin (concentrations of 1.4 μg/ml and 5.2 μg/ml, respectively). Expression of IFITM proteins was detected by Western blotting using an antibody against the HA tag (catalog number ab18181; Abcam), IFITM1 (catalog number HPA004810; Sigma), or IFITM3 (catalog number AP1153a; Abgent).

### IFITM1 localization.

The localization of IFITM-HA-tagged proteins was assessed using an anti-HA antibody conjugated to Dylight-550 (catalog number ab117502; Abcam). Coverslips were washed in phosphate-buffered saline (PBS) and adhered to microscopy slides using ProLong Gold with 4′,6-diamidino-2-phenylindole (DAPI) (ThermoFisher). Cells were imaged using microscopy after permeabilization in 0.25% Triton X-100 and fixed in 4% paraformaldehyde (PFA), and images were taken with a 63× objective. Expression of HA-tagged IFITM1 on nonfixed and nonpermeabilized cells was quantified by flow cytometry using an anti-HA antibody conjugated to Alexa Fluor 647 (catalog number 682404; BioLegend). Cells were washed in PBS and harvested by trypsinization. Cells were washed in 3% FBS–PBS and stained with antibody. Analysis was performed on an LSR Fortessa flow cytometer (BD Biosciences).

### RNA virus *in vitro* infections.

Transduced Vero cells were seeded at 2 × 10^5^ cells per well in 24-well plates. The following day, cells were infected with different paramyxoviruses: parainfluenza virus (PIV) strain rgPIV3 (MOI of 0.1) (42), measles virus (MV) strains rMV^rEdt^EGFP (43) and rMV^EZ^EGFP (44), mumps virus (L. J. Rennick and W. P. Duprex, unpublished data), Newcastle disease virus (NDV) strain rNDV-GFP-F0 (45), the orthomyxovirus influenza A virus PR/8/1934-EGFP (MOI of 1) (46), and the pneumoviruses respiratory syncytial virus B strain rHRSV^B05^EGFP (47) and strain A2 rgRSV (48) and human metapneumovirus (HMPV) strains NL/1/00-GFP (49) and HMPV NL/1/99-GFP (50). After 24 h, cells were fixed in 2% (vol/vol) PFA, and the percentage of infected cells was measured by detecting GFP expression using flow cytometry.

### HSV-1 *in vitro* infections.

HSV-1 C12, a variant that has a CMV IE1 promoter-enhanced green fluorescent protein (EGFP) cassette inserted at the US5 gene locus from pEGFP-C1 (Clontech), a kind gift from Stacey Efstathiou, was used for these experiments (51). Virus stocks were propagated and titrated on confluent BHK-21 cells.

HSV-1/GFP infection, at an MOI of 5 for A549 cells and at an MOI of 0.5 for MRC-5 cells, was determined by fluorescence microscopy at 7 h postinfection (hpi), unless stated otherwise, following fixation with 4% (vol/vol) PFA for 20 min and permeabilization using 0.3% (vol/vol) Triton X-100–PBS (10 min). Cells were washed with 100 μl of PBS-Hoechst solution (200 ng/μl; Life Technologies). The fixed cells were analyzed to determine the proportion of cells expressing GFP (Cellomics ArrayScan VTI; ThermoFisher), using the Target Activation bioapplication. Briefly, this method counts every cell on the plate by drawing a perimeter around each nucleus (detected by Hoechst staining) and calculates the percentage of these cells expressing GFP. Alternatively, flow cytometry was used to quantify HSV-1/GFP infection. Cells were washed in PBS and removed from the plastic using trypsin. Cells were washed again in PBS and fixed in 4% (vol/vol) PFA for 10 min at room temperature (RT). Cells were washed and resuspended in PBS and analyzed for GFP expression using a FACSCalibur instrument (Becton, Dickinson).

### Knockdown of IFITM1 using siRNA treatment.

MRC-5 cells were seeded in triplicate in 12-well plates at 6 × 10^4^ cells per well. The following day, cells were treated with either 5 μl of PBS (mock), 5 μl of IFN-α2a (PBL Interferon), 5 μM human IFITM1 SMARTpool siRNA (catalog number L-019543-00; Dharmacon), or 5 μM nontargeting pool siRNA (NT siRNA) (catalog number D-001810-10; Dharmacon). Transfections were carried out using the Dharmafect reagent according to the manufacturer’s guidelines.

### Confirmation of protein expressing using Western blotting.

Total protein was quantified by a bicinchoninic acid (BCA) assay (Thermo Scientific), and equal amounts of protein were loaded onto Mini-Protean TGX precast SDS-PAGE gels (Bio-Rad). Proteins were transferred onto nitrocellulose membranes using a TransBlot Turbo apparatus (Bio-Rad). Nitrocellulose membranes were blocked overnight using 5% (wt/vol) milk powder–PBS-Tween. Proteins were visualized with the following primary antibodies: human IFITM3 (rabbit anti-IFITM3, N-terminal amino acids 8 to 38, catalog number AP1153a; Abgent), human IFITM1 (rabbit anti-IFITM1, catalog number HPA004810; Sigma), and β-actin (rabbit anti-β actin, catalog number ab8227; Abcam), which was used as a loading control. All primary antibodies were visualized using species-specific horseradish peroxidase-conjugated secondary antibodies (Dako).

### Bioinformatic analysis.

Custom scripts (available upon request) were used to extract single variants in the IFITM1 locus of people in the 1000Genomes phase 3 cohort and people recruited in the following UK10K cohorts: UK10K_Neuro_Aberdeen, UK10K_Neuro_Asd_Gallagher, UK10K_Neuro_Edinburgh, UK10K_Neuro_Gurling, UK10K_Neuro_Iop_Collier, UK10K_Neuro_Muir, UK10K_Obesity_Gs, UK10K_Obesity_Twinsuk, UK10K_Rare_Hyperchol, UK10K_Rare_Neuromuscular, UK10K_Rare_Sir, UK10K_TwinsUK, and UK10K_ALSPAC. The resulting SNPs were analyzed using the Variant Effect Predictor tool (Ensembl), displaying results as one consequence per variant. Visualization of SNPs was performed using Protter (52).

### Mouse husbandry and phenotyping.

Background-matched 8- to 10-week-old wild-type or *Ifitm1*^−/−^ mice (Wellcome Trust Sanger Institute) (53), all of which were >95% C57BL/6, were supplied with food and water *ad libitum* and monitored daily for signs of illness. *Ifitm1*^−/−^ gene knockout (KO) mice were phenotyped through pipelines at the Wellcome Trust Sanger Institute, as described previously (54, 55). To investigate IFITM1 gene expression, 8- to 10-week-old BALB/c mice were obtained from Charles River (Bath, UK) and housed at the central biomedical sciences at Imperial College London. All animal experiments were performed in accordance with UK Home Office regulations and the UK Animals (Scientific Procedures) Act 1986 and reviewed by the Wellcome Trust Sanger Institute’s or Imperial College London’s Animal Welfare and Ethical Review Boards.

### RSV *in vivo* infection.

RSV strain A2 (a kind gift from P. Openshaw, Imperial College London) was grown in HEp-2 cells, and viral titers were determined by a plaque assay. Mice were infected intranasally with 5 × 10^5^ PFU under isoflurane anesthesia. Weight was measured daily to monitor disease severity. At day 7 after infection, lungs were removed, the smaller lobe was snap-frozen in liquid nitrogen for RNA extraction, and the remainder was homogenized by passage through 100-μm cell strainers (Falcon). The RSV viral load was measured by quantitative reverse transcriptase PCR (RT-PCR) for the RSV L gene using primers and probes previously described (56), with copy numbers determined using a curve and presented relative to micrograms of lung RNA. Lungs were homogenized with a rotor-stator homogenizer and centrifuged, and the supernatant was collected for cytokine analyses. Cytokines in lung homogenates were quantified by an enzyme-linked immunosorbent assay (ELISA) using duosets from R&D Systems.

### mCMV infections.

Smith strain mCMV was propagated *in vivo*, and virus stocks and viral loads in tissues of infected mice were quantified by a plaque assay, as previously described (27). Mice were infected with 3 × 10^4^ PFU of virus via the intraperitoneal route. IL-6 in organ homogenates was quantified using an ELISA (BioLegend). For *in vitro* infections, MEFs and bone marrow-derived myeloid cells were infected with mCMV, and virus production was quantified as previously described (27).

### Data availability.

GenBank accession numbers for the wild-type human genes cloned are MK288009 for IFITM1, MK288010 for IFITM2, and MK288011 for IFITM3.
